# S127. “HOW COULD THIS HAPPEN?”: PSYCHOSIS OR DEPRESSION AS A FACTOR IN DEATH BY SUICIDE

**DOI:** 10.1093/schbul/sby018.914

**Published:** 2018-04-01

**Authors:** James Overholser, Alison Athey, Eleanor Beale, Lesa Dieter, Craig Stockmeier

**Affiliations:** 1 Case Western Reserve University; 2 University of Mississippi Medical Center

## Abstract

**Background:**

Numerous factors play a role in the path of self-destruction that ends in suicide. The risk of suicidal behavior is increased when a depressed patient is also struggling with psychotic symptoms. Likewise, among adults with a psychotic disorder, suicide risk is related to depression, hopelessness, low self-esteem, social isolation and stressful life events. The present study was designed to examine the differential impact of major depression versus psychotic thinking on suicide risk in adults.

**Methods:**

Subjects:

The present study evaluated 104 adults who had died by suicide. Among these suicidal adults, 81 met diagnostic criteria for a Major Depressive Disorder at the time of their death, 10 met criteria for a psychotic disorder and 13 met criteria for the presence of both depression and psychosis.

Measures:

Structured Clinical Interview for DSM-Disorders (SCID: First et al., 1994) used informant interviews to evaluate the presence of major mental illness in the adult who died by suicide. Interviews were conducted with family members, gathering detailed information about the duration and severity of major mental illness.

Suicidal Actions Checklist (Dejong, Overholser, & Stockmeier, 2010) was used to gather information about recent stressors, previous suicide attempts and past hospitalizations for psychiatric problems. Prior research (Dejong & Overholser, 2009) has documented an adequate level of agreement for the Suicidal Actions Checklist when collected from suicide attempters as compared to family member informants.

Procedures:

Assessment procedures followed the guidelines for psychological autopsy research (Hawton et al., 1998), whereby family members were interviewed two months after the death of their loved one. In order to determine the most accurate diagnosis for each case, all records were reviewed by a psychiatrist, a clinical psychologist, a social worker, and a neuroscientist.

**Results:**

Because of the small number of patients with a psychotic disorder (with or without depression), these cases were combined into one group, and several non-significant trends are reported. As compared to suicide completers with a depressive disorder, the psychotic cases were more likely to be younger (t = 2.18, p < .05), unmarried (χ2 = 3.13, p < .08), unemployed at the time of their death (χ2 = 9.75, p < .01), and more likely to meet criteria for cannabis abuse (χ2 = 3.75, p < .06). Suicide completers with nonpsychotic depression were more likely to meet criteria for a comorbid diagnosis of alcohol abuse (χ2 = 4.36, p < .05) and often had alcohol in their system at the time of their death (χ2 = 4.35, p < .04). Despite the higher rate of personality disorders among the depressed completers (χ2 = 3.14, p < .08), the psychotic cases of suicide were more likely to have a chronic course to their symptoms (χ2 = 3.11, p < .08) with a history of prior psychiatric hospitalization (χ2 = 18.22, p < .01). Although not significant, when the three groups were examined separately, the depressed psychotic cases were more likely to have attempted suicide prior to the actual death by suicide (62%) compared to the depressed non-psychotic patients (37%) as well as the psychotic non-depressed cases (40%). Qualitative analyses will examine various patterns that may have direct links to suicidal urges.

**Discussion:**

Adults with a psychotic disorder have a more chronic condition that appears difficult to treat and tends to impair work or home functioning. The psychotic patients were less likely to rely on alcohol to lower their inhibitions about committing a suicidal act, suggesting other factors need to be addressed in prevention efforts. Patients may allow their psychotic thinking to guide their behavior, and when combined with depression can result in self-destructive actions.

